# Dataset for the spore surface proteome and hydrophobin A/RodA proteoforms of A.flavus

**DOI:** 10.1016/j.dib.2019.103817

**Published:** 2019-03-15

**Authors:** Mohammed Razeeth Shait Mohammed, Muthu Kumar Balamurgan, Rabbind Singh Amrathlal, Priyadharshini Kannan, Jeya Maheshwari Jayapal, Venkatesh Prajna Namperumalsamy, Lalitha Prajna, Kuppamuthu Dharmalingam

**Affiliations:** a Department of Proteomics, Aravind Medical Research Foundation, Dr. G. Venkataswamy Eye Research Institute, Aravind Eye Care System, Madurai, TamilNadu, India; b Cornea Clinic, Aravind Eye Hospital, Aravind Eye Care System, Madurai, TamilNadu, India; c Department of Ocular Microbiology, Aravind Eye Hospital, Aravind Eye Care System, Madurai, TamilNadu, India; d Department of Microbiology, Aravind Medical Research Foundation, Dr. G. Venkataswamy Eye Research Institute, Aravind Eye Care System, Madurai, TamilNadu, India

## Abstract

Fungal keratitis is a major sight-threatening corneal infection: and mycotic keratitis is more common in tropical parts of the world including India. *Aspergillus flavus* and *Fusarium* are the predominant causative agents of corneal infection. We extracted conidial surface proteins of *A. flavus* from saprophyte and clinical isolates and analyzed the proteins using high resolution mass spectrometry. The data revealed ecotype specific alteration in surface proteome since the proteome profile of the clinical isolates and saprophyte showed significant differences. Detailed examination of the mass spec data of RodA proteins extracted from polyacrylamide gels revealed the presence of two proteoforms of this protein. We also identified the mechanism of formation of these two isoforms. Detailed analysis of this data and the conclusions derived are described in the article, “Identification of the proteoforms of surface localized Rod A of *A. flavus* and determination of the mechanism of proteoform generation” [1].

**Table:** Specifications table

Subject area	
More specific subject area	Conidial surface proteome of *Aspergillus flavus*
Type of data	Figures,.xlsx files
How data was acquired	Tandem mass spectrometry (LC-MS/MS) using Thermo Easy nLC 1000 (Thermo, USA) coupled to Orbitrap Velos Pro mass spectrometer (Thermo, USA)
Data format	Raw, filtered, processed and analyzed,
Experimental factors	Dry spores of the isolates were extracted, and the total proteins were analyzed. RodA protein and its proteoforms were examined in detail.
Experimental features	Conidial surface protein prepared from the saprophyte (ATCC26) and two clinical isolates (CI 1698 and CI 1123) were pre-fractionated on 1D SDS-PAGE. Proteins in the gel pieces were processed, subjected to tryptic/gluC digestion and the extracted peptides after cleanup were analyzed in an Orbitrap mass spectrometer.
Data source location	
Data accessibility	Data is with this article
Related research article	Shait Mohammed MR, Balamurugan M, Amrathlal RS, Kannan P, Jayapal JM, Namperumalsamy VP, Prajna L, Kuppamuthu D: Identification of the proteoforms of surface localized Rod A of *Aspergillus flavus* and determination of the mechanism of proteoform generation. J Proteomics 2019, 193:62–70 [1]

**Table:** 

**Value of the data** • The data can be used for examining the in-depth profile of conidial surface proteins of *A. flavus* isolates. • The data demonstrates the difference in conidial surface proteome of saprophyte and clinical isolates. • The data can be used for examining the role of two proteoforms of RodA protein in the virulence of *A. flavus* • The data can be used for examining the mechanism of proteoform generation in fungi.

## 1 Data

**Fig. 1:**
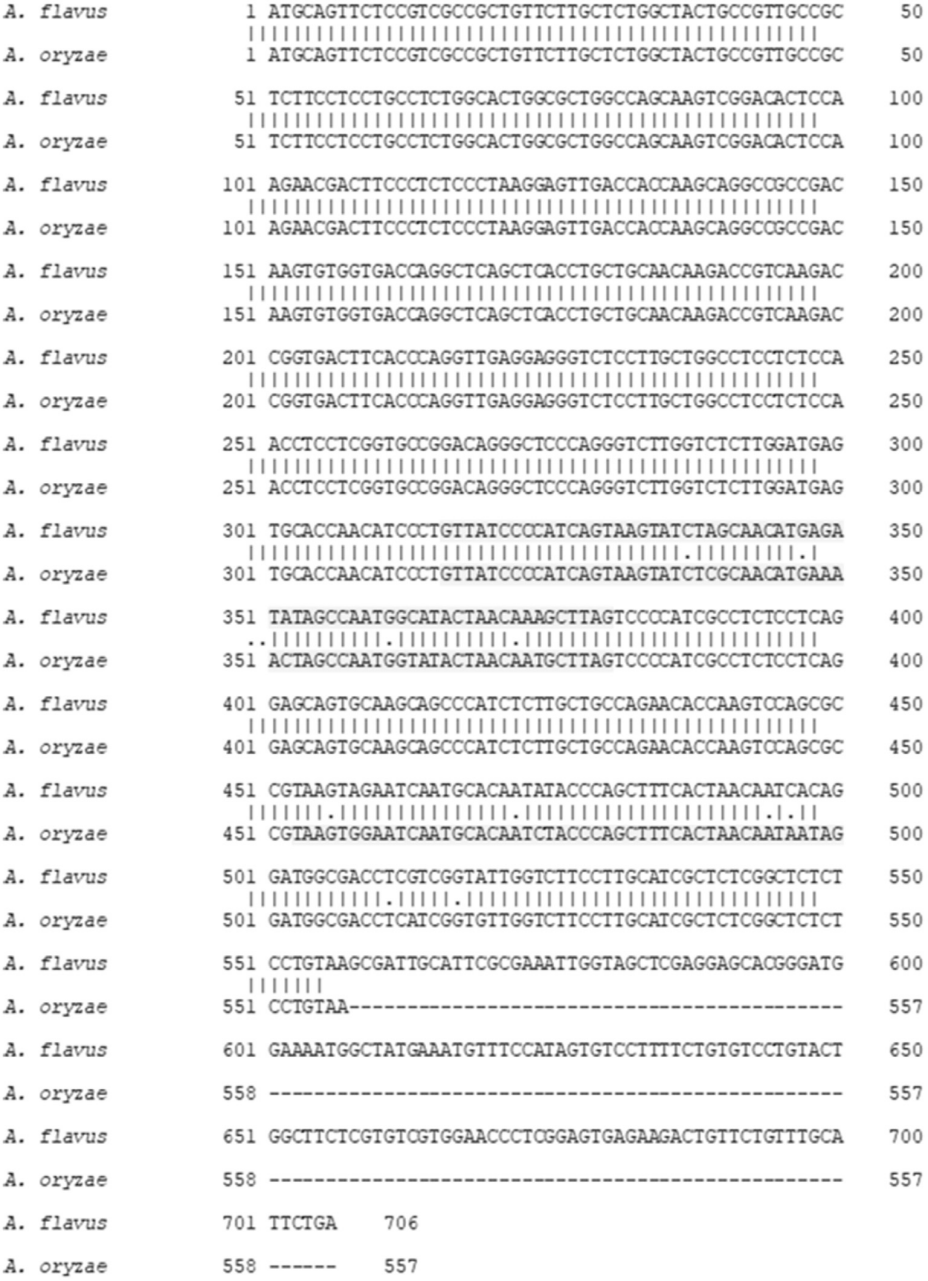
Pairwise alignment of *rodA* gene of *A. flavus* and *A. oryzae* transcripts. Intron one and intron two sequences are highlighted.

**Fig. 2:**
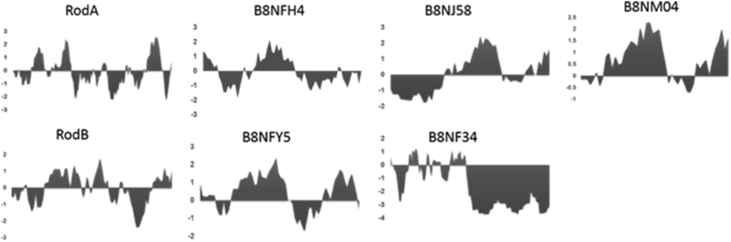
Hydrophathy plot for seven hydrophobins identified in the spore surface proteome.

**Fig. 3:**
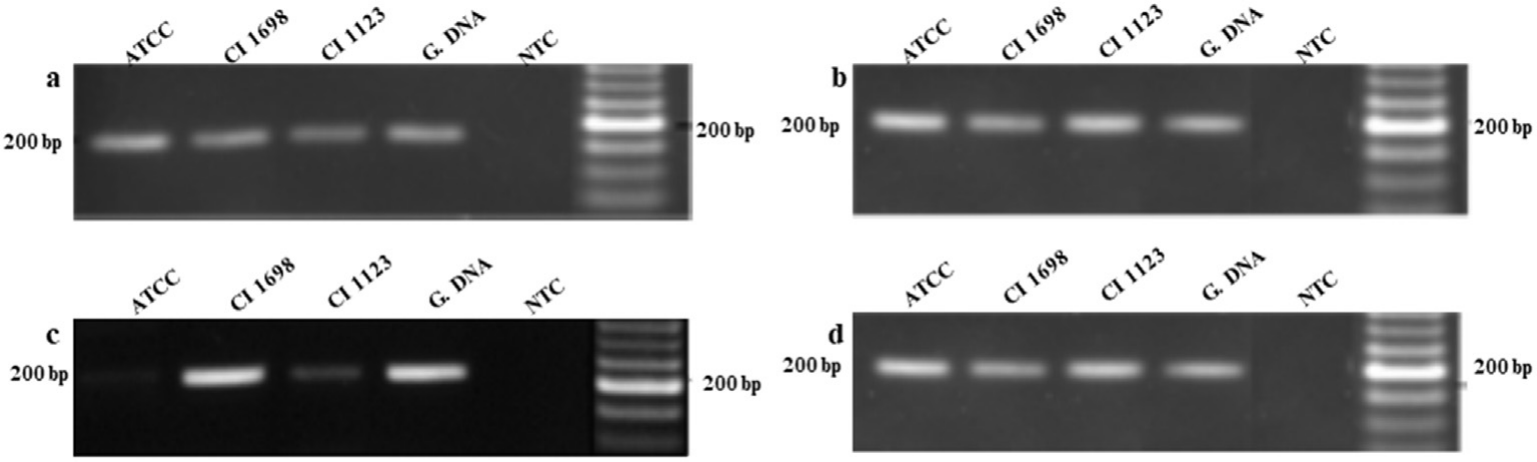
RT PCR analysis of *rodb* transcripts from cultures grown for 24 hrs and 40 hrs at wo different temperatures. a and b cultures grown at 30 °C or 37 °C respectively for 24 hrs. c and d cultures grown at 30 °C or 37 °C respectively for 40 hrs.

Pairwise comparison of the *rod*A gene of *Aspergillus flavus* and the related strain *Aspergillus oryzae* are shown in Fig. 1. Fig. 2 shows the deduced hydrophobicity plot of the RodA protein and Rod B proteins. RT-PCR data of the transcripts of *rodB* from mycelia grown at two different temperature is shown in Fig. 3. Spore surface proteins of saprophyte, CI1123 and CI 1678 are listed in Supplementary Tables 1–3. Supplementary Table 4 shows the pattern of conserved cysteins in RodA and Rod B proteins of *A. flavus.*

## 2 Experimental design, materials and methods

### 2.1 Preparation of samples for mass spectrometry

Previous report showed selective extraction of spore surface RodA protein of *Aspergillus fumigatus* by formic acid [2]. This procedure has been optimized as described below to avoid extraction of other surface proteins from spores. Spores were collected from CZA plates and mixed with 400 μl for 100% formic acid and incubated at 4 °C on ice for 1 h. After incubation spore suspension was centrifuged at 5000 rpm at 4 °C for 10 min, and then the supernatant was dried under a stream of nitrogen. The dried spore extract was resuspended in lysis buffer. Proteins were separated using 16% SDS PAGE and the proteins were visualized using Coomassie Brilliant Blue staining.

## 2.2 Mass spectrometry of spore surface proteins

Analysis of in gel-digested proteins was done as described below. In brief, samples were electrophoresed until the dye reached 1 cm in to the separating gels. Stained gel pieces were washed twice with water and completely destained by repeated incubation in 25 mM ammonium bicarbonate prepared in 50% acetonitrile. Gels were dehydrated using 100% acetonitrile followed by reduction and alkylation [1]. After three washes in 100 μl of 100 mM ammonium bicarbonate gel pieces were dehydrated using 100% acetonitrile. Dehydrated gel pieces were dried under vacuum and were rehydrated for 30 min on ice with 600 ng of trypsin (Invitrogen) and 300 ng of glu-C (Promega) in 5 μl of 100 mM ammonium bicarbonate in 10% acetonitrile. Tryptic were then extracted from gel pieces using 25 μl of 0.1% trifluoroacetic acid (TFA) in 60% acetonitrile and then with 20 μl of 100% acetonitrile. Extracted peptides were combined and desalted using C18 tips, and stored at 4 °C. Just before analysis, the peptides were suspended in 10 μl of 0.1% FA and analyzed using Obitrap Velos Pro mass spectrometer [3], [4]. The number of surface proteins identified in each strain were comparable, with 492, 469 and 477 proteins in ATCC (Supplementary Table 1), CI 1123 (Supplementary Table 2) and CI 1698 (Supplementary Table 3), respectively. Of these, only 289 proteins were common to all three isolates.

LC-MS/MS parameters were described in detail previously [3]. All MS/MS raw data acquired from Orbitrap Velos Pro Mass Spectrometer were analyzed by Proteome Discoverer v1.4 using Mascot (Matrix Science, London, UK; version 2.4.1.0) and the inbuilt Sequest HT algorithm. Both Sequest HT and Mascot were set up to search the database containing the complete human proteome including the isoforms downloaded from the Uniprot database on 31st July 2013 (141130 entries) and its Decoy database. In addition, Raw files were also searched using PEAKS software against *A. flavus* database from uniport. Protein products from mRNA with introns were identified using the *in vitro* translated protein sequence from the unspliced mRNA. *A. flavus rodA* gene sequence (NCBI gene ID 7910713) was downloaded from NCBI database and the sequence of unspliced and spliced mRNA were deduced, based on the data from closely related *A. oryzae rodA* sequence (Fig. 1). ExPASy Translate tool was used to convert the *rodA* mRNA sequences to amino acid sequence and used for the analysis of mass spectrometry data.

The genome of *A. flavus* encodes seven hydrophobin family proteins and their identity were confirmed by the presence of cysteine pattern and hydropathy plot (Fig. 2 and Supplementary Table 4). Data of RT PCR analysis of *rodb* mRNA was done as described in the main article [1] indicated the strain specific expression of *rodB* in cultures grown for 40 hrs at 30 °C (Fig. 3).

## Appendix ASupplementary data

Multimedia compenent 2 List of conidial surface protein identified from ATCC 26. Multimedia compenent 2

Multimedia compenent 3 List of conidial surface protein identified from CI 1123. Multimedia compenent 3

Multimedia compenent 4 List of conidial surface protein identified from CI 1698. Multimedia compenent 4

Multimedia compenent 5 Conserved cysteines pattern of seven hydrophobin. Multimedia compenent 5
