# Supplementary material for: Dataset for the spore surface proteome and hydrophobin A/RodA proteoforms of A.flavus
Source: Data Brief. 2019 Mar 15;23:103817. doi: 10.1016/j.dib.2019.103817 (PMC6660596; doi:10.1016/j.dib.2019.103817)
Supplement: Multimedia component 1 [file mmc1.docx]

We declare that we have no conflicts of interest.
