# Supplementary material for: Dataset for the spore surface proteome and hydrophobin A/RodA proteoforms of A.flavus
Source: Data Brief. 2019 Mar 15;23:103817. doi: 10.1016/j.dib.2019.103817 (PMC6660596; doi:10.1016/j.dib.2019.103817)
Supplement: Multimedia compenent 5 — Conserved cysteines pattern of seven hydrophobin. [file mmc5.xlsx]

**Supplementary Table 4: Conserved pattern of 8 cysteine residues in *A. flavus* hydrophobin gene**

| **Class I** | **CX5-7** | **CCX19-39** | **CX8-23** | **CX5** | **CCX6-18** | **C** |
| --- | --- | --- | --- | --- | --- | --- |
| Rod A | CX6 | CCX39 | CX17 | CX5 | CCX44 | C |
| Rod B | CX8 | CCX38 | CX10 | CX5 | CCX21 | C |
| AFLA_060780 | CX6 | CCX30 | CX23 | CX5 | CCX4 | C |
| AFLA_131460 | CX5 | CCX32 | CX6 | CX5 | CCX13 | C |
| AFLA_064900 | CX7 | CCX15 | Cx6 | CX5 | CCX8 | C |
| AFLA_094600 | CX7 | CCX16 | CX6 | CX5 | CCX9 | C |
